# Late Opacification of a Hydrophilic Acrylic Intraocular Lens

**DOI:** 10.4103/0974-9233.53374

**Published:** 2008

**Authors:** Muawyah D. Al-Bdour, Lana S. Dahabreh

**Affiliations:** From the Department of Ophthalmology, Jordan University HospitaJordan University Hospital, Amman, Jordan

**Keywords:** cataract surgery, intraocular lens, opacification

## Abstract

Cataract extraction and intraocular lens implantation is considered to be a safe procedure in most cases. However, the new advances in the surgical technique namely phacoemulsification and hence the increased use of foldable intraocular lenses have given rise to new complications including late opacification of intraocular lenses. In this case we report late opacification of a foldable hydrophilic acrylic intraocular lens and the surgical technique for its exchange.

Cataract extraction and intraocular lens (IOL) implant tation is considered to be a safe procedure in most cases. However, the new advances in the surgical technique namely phacoemulsification and the increased use of foldable intraocular lenses have given rise to new complications including late opacification of intraocular lenses. We report on such a case, the type of lens deposits detected, as well as management and prognosis.

## Case Report

**Figure 1:**
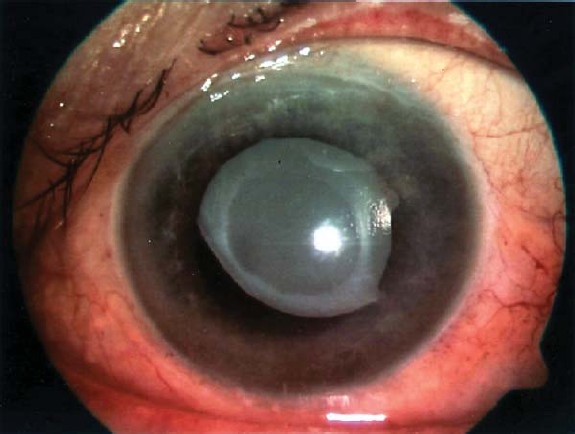
The opacified IOL before exchange as seen on slit-lamp examination.

A 60-year-old male hypertensive patient, who was on hemodialysis for chronic renal failure, underwent uneventful left phacoemulsification surgery and posterior chamber intraocular lens (IOL) implantation in February 2003. A foldable Aqua-Sense single-piece hydrophilic acrylic lens was implanted in the bag (Overall diameter 12.5 mm, Optic diameter 6.0 mm). His vision improved to 20/30 and he was doing well. Eye examination was normal apart from few macular drusen in both eyes. 20 months later the patient started to feel that the image was slightly dimmer in the left eye. The IOL was slightly cloudy but since the visual acuity was still unaffected intervention was postponed. The patient continued to have good vision till September 2006 when it deteriorated to light perception and the IOL cloudiness increased in intensity (Figure 1). Fundus examination became difficult.

He underwent exchange of the opacified IOL in September 2006, approximately 3 and half years after the original surgical procedure. It was technically difficult to remove the opacified IOL due to tight adhesions with the capsular bag. First we had to widen the pre-existing capsularrhexis using an MVR blade and vannus scissors. Viscoelastic material was then injected into the bag and a glide was inserted behind the IOL pushing it into the anterior chamber and out of the superior limbal incision. Anterior vitrectomy was performed because of limited posterior capsule rupture and a polymethylmethacrylate (PMMA) 6.5mm IOL was inserted in the sulcus.

**Figure 2:**
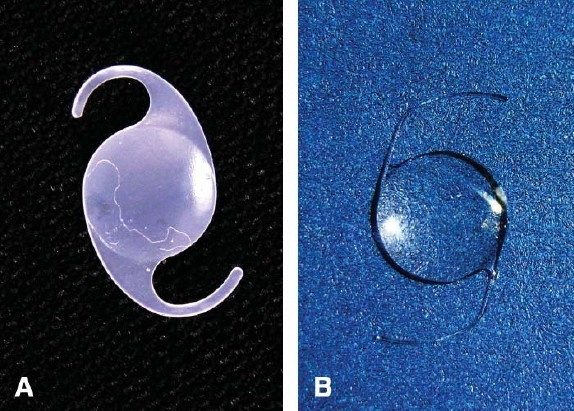
The explanted opacified IOL optic and haptics (a) is compared to a normal IOL (b).

The patient's vision improved to 20/60 on the first post-operative day and continued to be stable during regular follow-up visits. Examination of the explanted lens showed complete opacification of the optic and the haptics (Figure 2). Chemical analysis revealed traces of Calcium and Phosphate detected by Sodium Hydroxide and Potassium Hydroxide reagents respectively.

## Discussion

Several cases of late opacification (with patients usually becoming symptomatic during the second year or later) of hydrophilic IOLs have been reported in the literature.^1^–^3^ In the case of Aqua-Sense lenses it has been described to resemble nuclear cataract and is usually complete. Izak described two patterns of deposits as revealed by light microscopic and scanning electron microscope (SEM) analyses. Both irregular granular deposits on the external optical surfaces of the lens and multiple fine, granular deposits within the lens optic, distributed in a line parallel to the anterior and posterior curvatures of the optic, with a clear zone just beneath its external surfaces were exhibited simultaneously in Aqua-Sense lenses.^1^

There have been suggested causes for this late IOL opacification mostly related to Calcium deposits.^1^–^5^ Some report lens deposits that stained positive with alizarin red and the von Kossa method (for calcium). Energy dispersive X-R spectroscopy (EDS) also demonstrated the presence of calcium and phosphates within the deposits.^1^,^4^ Our explanted lens contained traces of Calcium and Phosphate detected by Sodium Hydroxide and Potassium Hydroxide reagents respectively.

Surgical explantation of IOLs is required in cases of vision threatening opacification. The technique is usually hazardous and challenging due to the tight adherence of the IOL to the capsular bag. Cutting the haptics before removal of the opacified IOL is sometimes required.

Complications include zonular dehiscence, rupture of the posterior capsule and corneal decompensation.^6^,^7^ Circumferential enlargement of the pre-existing capsulorrhexis is a critical step during surgery and would minimize traction on the capsule and hence the risk of zonular dehiscence or posterior capsule rupture. It was mandatory in our case as well.^7^

## Conclusion

Late opacification of hydrophilic IOLs is being increasingly reported, making long follow-up periods essential. Calcium deposits have been detected in almost all cases. Despite the hazards, surgical lens exchange is still the only available effective treatment for achieving visual recovery.

## References

[1] Izak AM, Werner L, Pandey SK, Apple DJ (2003). Calcification of modern foldable hydrogel intraocular lens designs. Eye.

[2] Trivedi RH, Werner L, Apple DJ, Pandey SK, Izak AM (2002). Post cataract–intraocular lens (IOL) surgery opacification. Eye.

[3] Joseph A, Dua H S (2002). Late opacification of SC60B-OUV acrylic intraocular lenses. British Journal of Ophthalmology.

[4] Chan Joong Kim, Sang Kyung Choi (2007). Analysis of Aqueous Humor Calcium and Phosphate from Cataract Eyes with and without Diabetes Mellitus. Korean Journal of Ophthalmology.

[5] Taboada-Esteve JF, Hurtado-Sarrio M, Duch-Samper AM, Cisneros-Lanuza A, Menezo-Rozalen JL (2007). Hydrophilic acrylic intraocular lens clouding: a clinicopathological review. Eur J Ophthalmol.

[6] Dagres E, Khan MA, Kyle GM, Clark D (2004). Perioperative complications of intraocular lens exchange in patients with opacified Aqua-Sense lenses. J Cataract Refract Surg.

[7] Voros GM, Strong NP (2005). Exchange technique for opacified hydrophilic acrylic intraocular lenses. Eur J Ophthalmol.

